# Supplementary material for: Patterns of Intron Gain and Loss in Fungi
Source: PLoS Biol. 2004 Nov 30;2(12):e422. doi: 10.1371/journal.pbio.0020422 (PMC532390; doi:10.1371/journal.pbio.0020422)
Supplement: Table S1 — Also available at http://genes.mit.edu/NielsenEtAl/. (4.3 MB ZIP). [file pbio.0020422.st001.zip › NielsenEtAl/html/32.html]

AN4678.1.NCU01936.1.MG02924.1.FG08914.1


```
 CLUSTAL W (1.82) Multiple Sequence Alignments - Introns Inserted


Sequence 1: NCU01936.1	672 aa
Sequence 2: FG08914.1	684 aa
Sequence 3: MG02924.1	668 aa
Sequence 4: AN4678.1	674 aa
Alignment Length: 725 aa
Number Identitical Residues: 257 aa
Alignment Score (without introns) 13310


MG02924.1 	MSSSSPLLEPKRFFRDTSPVDAAERTQSQFTYRQLSQLAAYSTSCPLRVISHIDLDAFYA
NCU01936.1	-MSSPPHMQS---FRDLSPAGAPGMKRSRFTYRHIQQLAASSTSCPLRVIAHLDLDCFYA
FG08914.1 	-MASSPLHRP---FGASSPLNEGGWRRSQFTYRQFSQLASSNTSNPLRVIAHIDLDAFYA
AN4678.1  	-MPLSPEPPG----SPPNSSNDSLKRHSRYTYRHFQLLRQASPASPLRVIAHIDLDAFYA
          	  . .*           .. .     :*::***::. *   ..: *****:*:***.***

MG02924.1 	QCEMVRLGVDEDRPLAVQQW2QGLIAVNYPARKFGISRHCAIDDALKLCPELQVQH1VAT
NCU01936.1	QVEMVRLGIPDDKPVAVQQW2QGLIAVNYPARSFGIGRHCNVSEAKKLCPDLIHQH~VAT
FG08914.1 	QCEIVRLGIPEDRPLAVQQW2QGLIAVNYAAREYGIGRHCNVEEAKKLCPELIAQH~VAT
AN4678.1  	QCEMVRLGTPRERPLAVQQW~DSLIAINYPARKFGITRMISAKEAKAKCADIVLQH~VAT
          	* *:****   ::*:***** :.***:**.**.:** *    .:*   *.::  ** ***

MG02924.1 	WREGDN-TWAYRGDAAANIATDKVSLDPYRLQSRTILALIKECLP---------------
NCU01936.1	WREGDA-KWDYHPDAAANMATHKVSLDPYRLESRKILAIIKEELP---------------
FG08914.1 	WREGDD-KWAYRDDAAANIVTDKVSLDPYRLQSRKILACIKDALP---------------
AN4678.1  	FREGEGGKWAYREDAWKNIGTDKVCLDPYRAESRKILGTIKRELSKCYSELNGDLSGLGL
          	:***: ..* *: **  *: *.**.***** :**.**. **  *... :. ... :. . 

MG02924.1 	-----PHQCKVEKASIDEVFIDLSANVHSKLIERFPELASPPPGADAQARLPLPPVSALD
NCU01936.1	-----PHLQKVEKASIDEVFMDLSAQVHTILLERFPELANPPPYDDPTEMLPMPSITALD
FG08914.1 	-----ADVQKVEKASIDEVFLDLSSQVRSILLERFPELSNPPPYDDPTEKLPLPSIAALD
AN4678.1  	SSQIKLQEALVEKASVDEVFIDLSPLVFGVLLQRYPEMREKPHGDDRVALLPCPPTTALE
          	::. . .   *****:****:***. *   *::*:**: . *   *    ** *. :**:

MG02924.1 	WQ-ADALIELDETETETDDPDWDDIALLIASEITRDIRQTIFTRLKYTCSAGVAGNKLLS
NCU01936.1	WQ-ADALVDLPDENAELEDPDWDDVAILIGSEIVRKVRIAIKDKLGYTCSAGVACNKLLS
FG08914.1 	WQ-TDALIDLNEE-QEAIDPDWDDVAILIGSEIVRKVRSEVRQKLGYTCSAGVASNKLLS
AN4678.1  	WDTEDCLVDLDENETEVDDPDWDDVAMLIGAEIVRSVRTAVWNSLSYTCSAGIAKNKMMA
          	*:: *.*::* : . *  ******:*:**.:**.*.:*  :   * ******:* **:::

MG02924.1 	KLGSAHKKPNQQTVIRGRAVRKFLSDFKFTKIRNLGGKLGDQVVAAFGTESVSDLLEVPL
NCU01936.1	KLGSAYRKPNQQTVLRNRSIQHFLSDFKFTKMRNLGGKLGEQISQMFHTDTVKDLLSASV
FG08914.1 	KLGSAYKKPNKQTVVRNRAVSAFMAGFKVMKLRNMGGKLGEQIVSTFNTENVTELLEVPL
AN4678.1  	KLGSATNKPNKQTVVRNRAIQNFLSGFKFTKIRMLGGKLGDQVTAMFGTEQISELLKVSL
          	***** .***:***:*.*::  *::.**. *:* :*****:*:   * *: :.:**...:

MG02924.1 	DQLKLRLGEESTALWVYNTIRGIDNSEVNSRTQIKSMLSAKSFRPSIHSVDQANKWLRIF
NCU01936.1	EQLKSKLG-DDTGVWVYNTVRGIDTSEVNPRVQIKSMLSAKSFRPSITSFEQAVRWLRIF
FG08914.1 	SSMKAKLG-HDTGFWVFNTIRGIDTSEVNPRTQIKSMLSAKSFRPTINSSEQATRWLRIF
AN4678.1  	EQFRAKLN-DDTAVWLYGIIRGEDKSEVNPRTQIKSMLSAKSFRPSINTLDQADKWLHIF
          	..:: :*. ..*..*::. :** *.****.*.*************:* : :** :**:**

MG02924.1 	SADIFSRLVEEGVLENKRRPKTMNLHHRHNGQSKARSCPIPQGVRVLDDSVLYELAQNLL
NCU01936.1	AADIFSRLVEEGVLENKRRPKTINLHHRHGTQTRSRQGPIPQG-RKLDEESLLELARNLL
FG08914.1 	AADIFARLVEEGVLENKRRPKTMNLHHRHEGQVRSRQVPIHQG-RALDEECLFELAKDLL
AN4678.1  	AADIYGRLVEEGVLEHKRRPKTIALHHRQGAQVKSRQLPIPGS-ATIDEDLLFELGKTLL
          	:***:.*********:******: ****:  * ::*. **  .   :*:. * **.: **

MG02924.1 	RQIVMEGK-VWPCDNLSLNVSGFEDGITGNMGIGGFLVKGEEAQGLRSVSNRDNPAGHQE
NCU01936.1	SQIVSEGQ-VWPCSNLSLSVGGFEDGVTGNMGIGAFLLKGEEAQASKTGSGTATVDSETE
FG08914.1 	SQIIAEGRGVWPCANLSLSVGGFEDGVKGNMGIGAFLVKGEEAEALRLSN-SESRPSSAG
AN4678.1  	RQIAADGA-AWPCANISMSVGGFEDGTSKNQAIDGFLLRGDQARNMASSSSSRVHVADEV
          	 **  :*  .*** *:*:.*.***** . * .*..**::*::*.     ..     .   

MG02924.1 	TQQQQHHDSKRRKINGGGLHRFFGKATQQRESSSGPGEDDEYTAKAEGSKRRSQDE----
NCU01936.1	IRP----AEKRRRLDNGGIERFFAKRELSHGANPGLGSDDTAISGQSGQRLGTLKEEVMS
FG08914.1 	PEP----LAKKRRVEDSGIQRFFAKRSSTD--HDGSSSNKSPTPNEKTTTVRTLSEDAEK
AN4678.1  	PAEHQP-IEKRRKVEDSGLRRFFNKPSETD--GPGHSFDKGHMADTPDINVDNAQLDD--
          	    .    *:*:::..*:.*** *         * . :.   .        . . .   

MG02924.1 	---------LHPTDAQAEESMQEAIGS---------------------SCPRCKDVFFNT
NCU01936.1	---------GEPAGSEGVGKGDEVYNGKSESEP---------GPIITLTCSRCN-ASLDS
FG08914.1 	SLSYVTNYGHEARHGSNISSHQFTVSSRHATAPSTDTDHGEQSSIDPLKCSRCK-ANFAD
AN4678.1  	---------HVASDGEIEQVHS---KS--------------------FRCPRCG--RYIN
          	           .  ..     .    .                      *.**       

MG02924.1 	PEELQDHLDWHLAKDLQDEEERGASAFATRRQPTSAAPPAKPRGRGRPPKGAAKPKTDPG
NCU01936.1	PEELQSHQDWHFAKELQEQE-RVSQTFVNQPSASSSRAGNQKSTSTMPKRQGRPKKVERG
FG08914.1 	PEALQSHEDWHMAKDLQDEE-RVNPTFAERQSTTRNTAPKTQGTAS---RRGRGGKLEQG
AN4678.1  	CGEEDEHNDWHFAKDLETQERQAARSSQAAQLPNRAAS----GSRSKPARGGRGGKSEKG
          	    :.* ***:**:*: :* :   :      ..   .         . : .   * : *

MG02924.1 	QRRLRFD
NCU01936.1	QSKLKFG
FG08914.1 	QSRLKFG
AN4678.1  	QTRLTFG
          	* :* *.
```
